# The Role of 4-Phenylbutyric Acid in Gut Microbial Dysbiosis in a Mouse Model of Simulated Microgravity

**DOI:** 10.3390/life12091301

**Published:** 2022-08-24

**Authors:** Shama Shama, Rizwan Qaisar, Naveed Ahmed Khan, Isfahan Tauseef, Ruqaiyyah Siddiqui

**Affiliations:** 1 Department of Microbiology, Hazara University, Mansehra 21300, Pakistan; 2 Department of Basic Medical Sciences, College of Medicine, University of Sharjah, University City, Sharjah 27272, United Arab Emirates; 3 Cardiovascular Research Group, Sharjah Institute for Medical Research, University of Sharjah, Sharjah 27272, United Arab Emirates; 4 Department of Clinical Sciences, College of Medicine, University of Sharjah, University City, Sharjah 27272, United Arab Emirates; 5 College of Arts and Sciences, American University of Sharjah, University City, Sharjah 26666, United Arab Emirates

**Keywords:** gut microbiome, microgravity, hindlimb unloading model, novel metabolites, ER stress, muscle weakness, gut dysbiosis

## Abstract

The altered gut microbes of astronauts during space travel may contribute to health issues after their return to Earth. Previously, an association between the elevated endoplasmic reticulum (ER) stress and gut microbial dysbiosis has been described. Herein, we induced gut microbial changes in mice under a simulated microgravity environment in an established model of hindlimb unloaded (HU) mice. The intestinal metabolomic profiles under microgravity conditions using the HU model were examined, along with the potential role of 4-phenylbutyric acid (4-PBA), a potent ER stress inhibitor. For a microgravity environment, the mice were suspended in special cages individually for three weeks. Mice were sacrificed, and gut dissections were performed, followed by amplicon sequencing analysis of bacterial species via DNA extraction and 16S rRNA analysis. The results indicate that the gut bacterial communities of mice differed under gravity and microgravity conditions. Principal component analyses revealed differences in the bacterial community structure in all groups. Around 434 operational taxonomic units (OTUs) were specific to mice seen in controls, while 620 OTUs were specific to HU mice. Additionally, 321 bacterial OTUs were specific to HU mice treated with 4-PBA. When the relative abundance of taxa was analyzed, Bacteroidetes dominated the gut of control and HU mice treated with 4-PBA.. In contrast, the untreated HU mice were dominated by Firmicutes. At the genus level, a reduction in beneficial species of *Akkermansia* and *Lactobacillus* was observed in HU but not the unloaded–treated and control mice. Furthermore, an increase in the relative abundance of *Lachnospiraceae* and *Enterorhabdus*, associated with inflammation, was observed in HUmice but not in controls and unloaded-treated mice. Following treatment with 4-PBA, the ratio of Firmicutes to Bacteroidetes was restored in unloaded–treated mice, comparable to controls. Of note, beneficial microbes such as *Akkermansia* and *Lactobacillus* were observed in unloaded–treated mice but not or in lesser relative abundance in HU mice. Nonetheless, microbial diversity was reduced in unloaded–treated mice compared to controls, and future studies are needed to mitigate this finding. These may comprise the addition of pre-/pro- and postbiotic species in the diet to increase microbial diversity. Overall, the findings suggest that 4-PBA, a potent ER stress inhibitor, may have therapeutic value in treating patients on prolonged bed rest or astronauts during spaceflight.

## 1. Introduction

The microorganisms that reside in the gastrointestinal tract or the gut microbiome are well known to interact with the immune system and affect human health [1]. Furthermore, the gut microbiome is thought to offer immunity against pathogens and may protect against various diseases such as cancer, metabolic disorders, and obesity, to name a few [2,3,4,5,6]. The role of the gut microbiome under microgravity environments during space travel and its impact on astronaut health needs further comprehension and is the subject of this study [7,8].

The gut microbes of the astronauts are altered during space travel, and this may contribute to the health issues that are faced following space travel [7,8,9]. The space and microgravity environment can expose astronauts to various detrimental effects, including physiological effects, as well as other multiple stressors with the potential to affect human performance and health. These conditions include radiation, side effects of medications, fluid shifts, noise, hypoxia, hypercapnia, decompression, dietary restrictions, and sleep deprivation [10]. Furthermore, microgravity may induce skeletal muscle disorders comprising muscle force decrease, atrophy, and fiber-type shift [11].

In this study, we utilized the hindlimb unloaded (HU) mouse, which is an established ground-based *in-vivo* model of microgravity [12]. The HU model mimics several physiological changes during space travel and prolonged bed rest including blood redistribution, headward fluid shift, insufficient oxygen, a reduced blood supply to the gastrointestinal tract [13]. Previous studies report an association between altered gut microbiota and cellular stress. Specifically, an increased protein dysregulation by the endoplasmic reticulum (ER), a condition called ER stress, has emerged as a candidate driver of gastrointestinal (GI) dysbiosis [14]. Furthermore, these mice also show intestinal dysbiosis and skeletal muscle decline along with elevated ER stress. Therefore, we investigated the intestinal metabolomic profiles under microgravity conditions using the HU model and examined the potential role of ER stress in intestinal dysbiosis. In particular, the role of 4-phenylbutyric acid (4-PBA), a well-known ER stress inhibitor known to enhance protein folding and restrain ER stress in vitro as well as in vivo, was investigated [15,16,17]. 4-PBA is provides protective effects in a wide array of diseases, including cancer, cystic fibrosis, thalassemia, type 2 diabetes mellitus, amyotrophic horizontal sclerosis, Huntington’s disease, Alzheimer’s disease, and Parkinson’s disease [18,19]. We hypothesize that 4-PBA may play a role and mitigate intestinal dysbiosis in HU mice by inhibiting ER stress. It is anticipated that the results from this study can be utilized as a model for further studies in humans under bed rest or during spaceflights. Future studies are warranted in order to prevent different conditions related to microgravity and stress causing gut dysbiosis during space travel, and pre-pro and postbiotics can be developed as treatment options for astronaut/human health.

## 2. Materials and Methods

### 2.1. Hindlimb Unloading (HU) Mouse Model

For the HU model, 4-month-old male c57BL/6j *wild-type* mice were used. This age was chosen because mice at this age are equivalent to approximately 30-year-old humans [20], which corresponds to the average age of astronauts in flight and includes a sufficient proportion of human patients with prolonged bed rest [21,22]. Mice were randomly divided into three groups (*n* = 3–5 mice/group), i.e., (i) the untreated control group (untreatedground-based mice without mechanical unloading), (ii) the HU mice that were treated with 4-PBA (100 mg/Kg/d via intraperitoneal injection) [23], and (iii) the unloaded group treated with phosphate-buffered saline (PBS) as a vehicle. Mice were maintained in controlled environments (20 ± 1 °C, light/dark periods of 12 h each) with food (standard chow diet for mice) and water *ad libitum* for three weeks. Mice in the unloaded group were tied to their tails with a string, and the other end was tied to the top of the cage (one mouse/cage) in specially designed cages [24]. At the end of the experiment, the mice were euthanized by cervical dislocation, and all organs, including intestinal tissues were immediately dissected and stored at −80 ° C for further analysis. All protocols for animals were sanctioned and approved by the Animal Care and Use Committee of the University of Sharjah) (letter no. ACUC-19-05-05-01) according to recognized international standards.

### 2.2. Extraction of DNA

Amplicon sequencing studies were performed by extracting DNA from the intestinal muscle, as described earlier [25]. In short, each sample was incubated with 1 mL of preheated lysis buffer (20 g of sodium dodececylsulphate per liter, 0.1 M Tris-HCl, 0.15 M sodium chloride, 25 MM EDTA), and pH was brought to 8.5. The samples were then added to 10 μM of proteinase K (10 mg per mL) and conditioned at 65 °C for 60 min. After centrifugation at 12,000× *g* for 10 min, the supernatant was first extracted with phenol/chloroform/isoamyl alcohol in the ratio of 25:24:1 (rev/rev/rev) and then chloroform/isoamyl alcohol in the ratio of 24:1 (rev/v). DNA was then deposited using potassium acetate (3 M, pH 5.5) and 95% ethanol, followed by centrifuges at 15,000× *g* for 10 min. The sludge was washed twice with 70% ethanol and then dissolved in 0.4 mL of the buffer (10 mM Tris, 1 mM EDTA). The samples were then incubated with 10 mg RNASA for 30 min at 37 °C to remove the residual RNA. The protein was then removed by extraction with chloroform/isoamyl alcohol. Finally, the upper layer was collected in a tube containing 2.5 vol of ethanol (second precipitation), and the DNA was precipitated and centrifuged at 15,000× *g* for 10 min. The DNA pellet was washed twice, dried, and then resuspended in 0.2 mL dH_2_O. DNA concentration and purity of were elucidated using a 1% agarose gel.

### 2.3. Sequencing of Bacterial 16 rRNA

Intestinal DNA was processed to sequence the hypervariable area of the gene 16S rRNA using primers 341F-CCTAYGGRBGCASCAG; 806R-GGGACTACNNGGTTATTAAT, as previously described [26,27,28]. PCR reactions were accomplished via the Phusion High-Fidelity PCR Master Mix (New England Biolabs^®,^ Ipswich, MA, USA). Then, a loading buffer containing the same volume of SYBR green was added, followed by gel electrophoresis on a 2% agarose gel. PCR products were cleaned with the Qiagen Gel Exportation Kit (Qiagen, Hilden, Germany). The sequence libraries were created using the NEBNext Ultra^TM^ DNA library set for Illumina, quantification using Qubit and Q-PCR, and analysis using the Illumina platform^®^, San Diego, CA, USA. Finally, the library was sequenced on the Ion S5TM XL platform and it generated one-sided readings of 400 p.m./600 p.m. The amplicon was sequenced on the Illumina two-way platform to acquire raw readings of the 250 p.n. pair ends. Qualitative filtering of raw tags was accomplished under certain filtering conditions to obtain high-quality clean tags according to the Qiime (V1.7.0) quality control process (http://qiime.org/scripts/split_libraries_fastq.html accessed on 1 February 2022) used for subsequent analysis.

### 2.4. Data Analysis

As the sequencing resulted in some “dirty data”, the raw data were combined and filtered to produce clean data to make the analysis of the information accurate and reliable. Then, on the basis of effective data, the clustering of operational taxonomic units (OTU) was carried out. Briefly, one-sided readings were assigned to patterns. Raw readings were filtered under specific filtering conditions to obtain high-quality clean readings according to the Cutadapt quality control process (V1.9.1, http://cutadapt.readthedocs.io/en/stable/ accessed on 1 February 2022) [29]. Next, the reads were correlated with the reference Silva database v138.1 (https://www.arb-silva.de/ accessed on 1 February 2022) [30] using the UCHIME algorithm (http://www.drive5.com/usearch/manual/uchime_algo.html accessed on 1 February 2022) [31] to detect chimeric sequences and then remove chimeric sequences [32] to obtain clean reads. Analysis of sequences was accomplished using the Uparse software (Uparse v7.0.1001, http://drive5.com/uparse/ accessed on 1 February 2022). Sequences with ≥97% similarity were assigned to the same OTUs. For each sequence, a Silva database established using the Mothur algorithm was utilized for the annotation of taxonomic details. To determine the phylogenetic relationship of different OTUs, as well as the difference between species prevalent in different samples (groups), multiple sequence alignments were completed using MUSCLE software (version 3.8.31, http://www.drive5.com/muscle/ accessed on 1 February 2022) [33]. Each OTU was sequenced, and taxonomic annotation was performed to obtain the corresponding taxa information and taxa-based abundance distribution. In addition, OTUs were analyzed for alpha diversity analysis and beta diversity analysis, and Venn diagrams were created to elucidate sample abundance and uniformity information, common and unique OTU information between different groups or samples, and more. Principal component analysis was used to know the similarity and dissimilarity matrices of microbiota among the three studied groups (control, unloaded, and unloaded–treated groups).

## 3. Results

Diversity of microbial communities across the three groups:

### 3.1. Interspecific Varieties in Bacterial Intestine Communities

**Figure 1:**
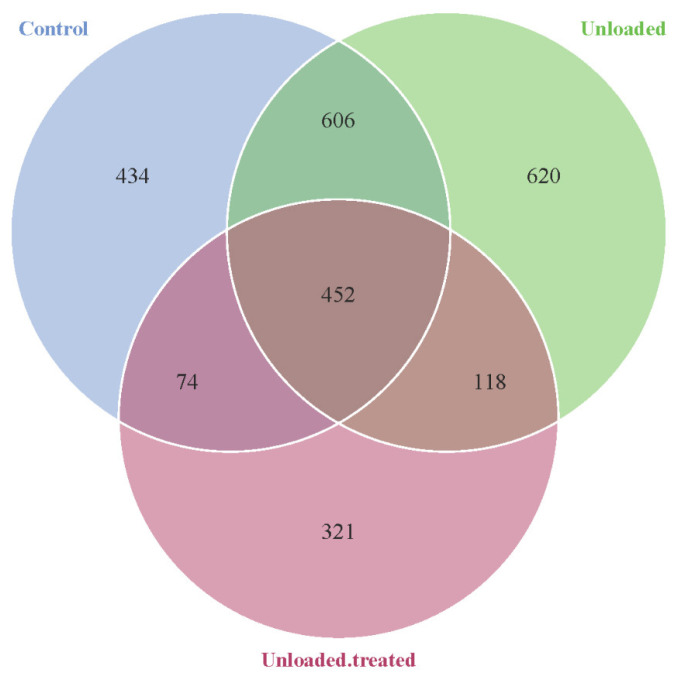
Venn diagram showing the distribution of bacterial OTUs between mice kept under gravity conditions (control) and mice kept under a microgravity environment (unloaded) and mice kept under a microgravity environment and treated with 4-PBA (unloaded–treated) according to the 16S rRNA gene sequence analysis.

Different gut OTUs were observed in the different groups of mice: controls, unloaded, and unloaded–treated, as depicted in Figure 1. In around 434 bacterial communities, OTUs were specific to the mice in ground-based controlled conditions, while in 620 bacterial communities, OTUs were specific to HU mice in simulated microgravity conditions. Additionally, 321 bacterial OTUs were specific to unloaded–treated mice. We found 606 bacterial OTU intestinal communities in common between the controls and unloaded mice, 118 bacterial OTUs in common between the unloaded and unloaded–treated mice, and 74 bacterial OTUs common between controls and unloaded–treated mice. On the other hand, there were 452 intra-section areas sharing common bacterial communities of OTUs between all groups.

### 3.2. Relative Abundance of Taxa

**Figure 2:**
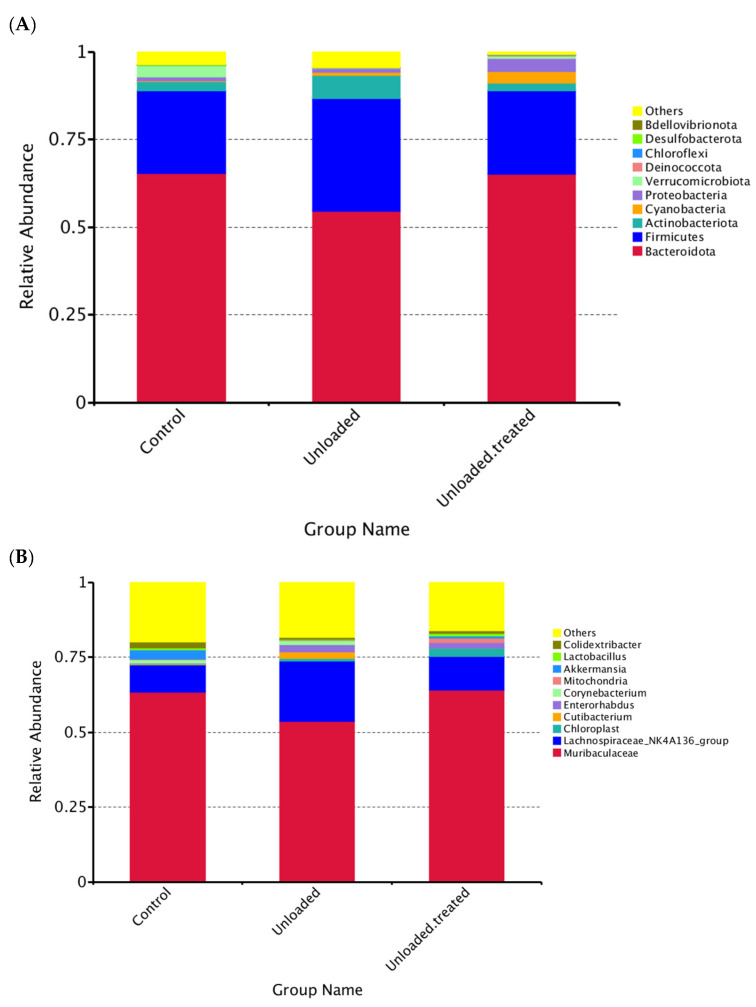
Distribution of dominant phyla (**A**) and genus (**B**) in mice under gravity environment (labeled as control) and under a microgravity environment (labeled as unloaded) and under a microgravity environment and treated with 4-PBA (unloaded treated).

The taxonomic annotation data revealed the top 10 taxa for each group of mice at different taxonomic levels (phylum, class, order, family, genus), which were assigned to form a distribution histogram of the relative abundance of taxa for visualization with comparative taxa with a high relative abundance and the proportion of each sample at different taxonomic levels, as depicted in Figure 2.

### 3.3. The Relative Abundances of Taxa at the Phylum Level

The relative abundances of taxa within phyla are shown in Figure 2A. “Other” indicates the total relative abundance of phyla other than the first 10 phyla. Interestingly, among phyla, *Bacteroidota* was the most prevalent phylum, being found in both controls and unloaded–treated mice and were around 65%. In contrast, *Firmicutes* had the highest abundance at around 48% in unloaded mice as compared to controls and unloaded–treated, which had a 27% equal abundance of *Firmicutes*. The relative abundance of *Bacteroidota* slightly differed between the three groups (Figure 2A).

Overall, the relative abundance of the top 10 phyla in mice in controls was in the order of *Bacteroidota, Firmicutes, Actinobacteria, Cyanobacteria, Proteobacteria, Verrucomicrobiota, Desulfobacterota*, and others. However, the relative abundance of the top 10 phyla in unloaded mice were in the order of *Bacteroidota, Firmicutes, Actinobacteria, Cyanobacteria, Proteobacteria, Deinococcota, Verrucomicrobiota, Campilobacterota*, and others. In contrast, the relative abundance of the top 10 phyla in unloaded–treated mice was in the order of *Bacteroidota, Firmicutes, Actinobacteria, Cyanobacteria, Proteobacteria, Verrucomicrobiota, Deinococcota, Bdellovibrionoa,* and others (Figure 2A).

### 3.4. The Relative Abundances of Taxa on the Level of Genus

At the level of the genus, the distribution of the genus *Muribaculaceae* between controls versus unloaded and unloaded–treated mice was significantly different. Both controls and unloaded–treated mice had the same ratio of the genus *Muribaculaceae* in contrast to unloaded mice, which had less distribution of genus *Muribaculacea.* The relative abundance of *Lachnospiraceae* was increased in relative abundance in unloaded mice compared to unloaded–treated and controls. Moreover, *Akkermansia* were not observed in unloaded mice and were present in controls as well as unloaded–treated mice, albeit with lesser relative abundance. Similarly, *Lactobacillus* were not observed in unloaded mice but were present in unloaded–treated mice and controls. Of note, *Cutibacterium* were observed in unloaded mice but not in unloaded–treated mice and controls. *Enterorhabdus* were observed in greater relative abundance in unloaded mice and in unloaded–treated mice but were minimal in relative abundance in controls (Figure 2B).

### 3.5. Analysis of the Principal Component Analysis (PCA) Test

**Figure 3:**
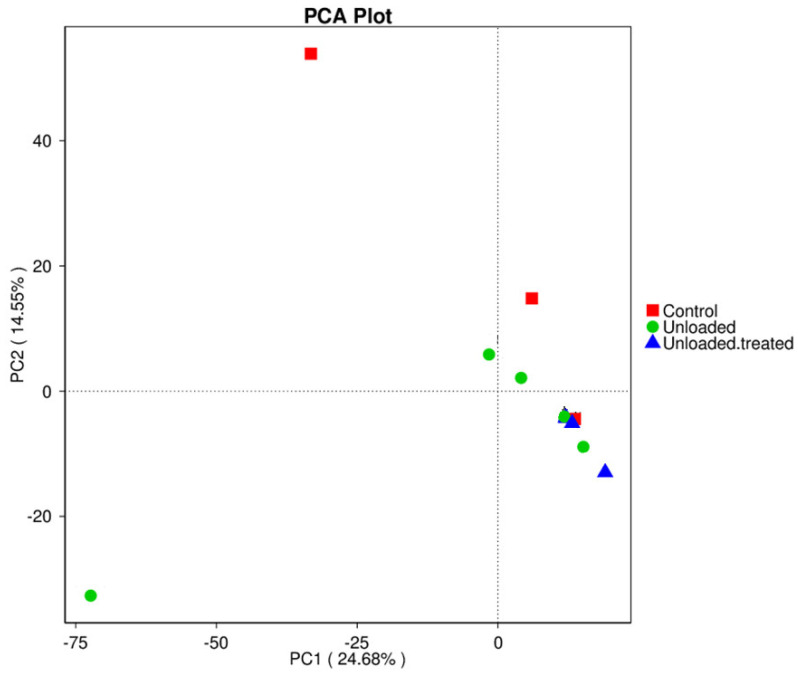
Principal component analysis (PCA) for the differences in microbial communities between mice under gravity environment (control), mice under microgravity environment (unloaded), and mice under microgravity environment and treated with 4-PBA (unloaded–treated).

In the principal component analysis (PCA) test, differences in the bacterial diversity between controls, unloaded mice, and unloaded–treated mice were elucidated and are presented in Figure 3. The data revealed that when major factors PC1 and PC2 were compared, the contribution of PC1 was 24.68%, while the contribution of PC2 was 14.55%. These results indicate that the groups exhibited differences in bacterial community structure.

### 3.6. Beta Diversity of Bacterial Community Composition

**Figure 4:**
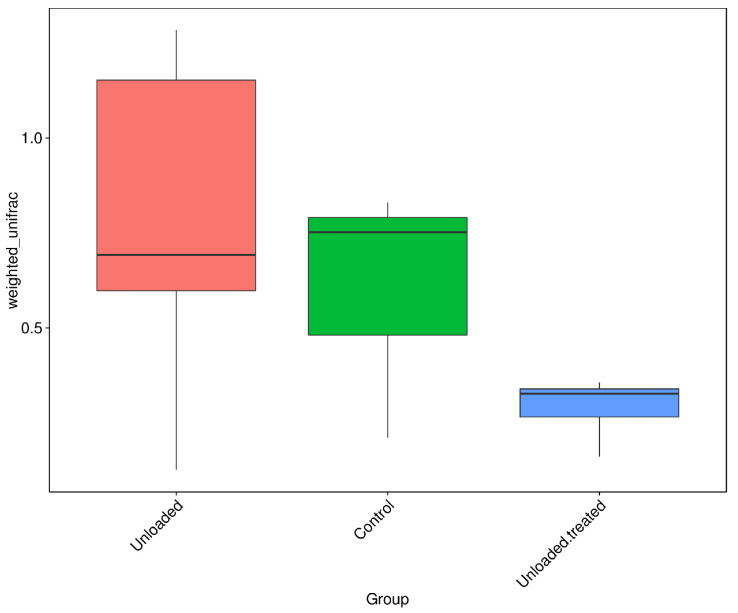
Boxplots based on Weighted Unifrac distance. Controls and unloaded mice revealed a larger closing cluster of beta diversity as compared to unloaded–treated mice, indicative of less variation in bacterial diversity communities between them. The intra-sample variation among controls was larger with less intra-sample variation within unloaded–treated mice.

Beta diversity represents the explicit comparison of microbial communities on the basis of their composition. The closing cluster of intra-sample variation of bacterial diversity is shown in Figure 4. A boxplot was generated to show the difference in beta diversity indices between groups. Wilcox and Tukey tests were performed for analysis of the significance of the difference between groups. Controls and unloaded mice revealed a larger closing cluster of beta diversity as compared to unloaded–treated mice, indicative of less variation in bacterial diversity communities between them. The intra-sample variation among controls was larger with less intra-sample variation within unloaded–treated mice.

### 3.7. Difference of Alpha Diversity Indices between Groups

**Figure 5:**
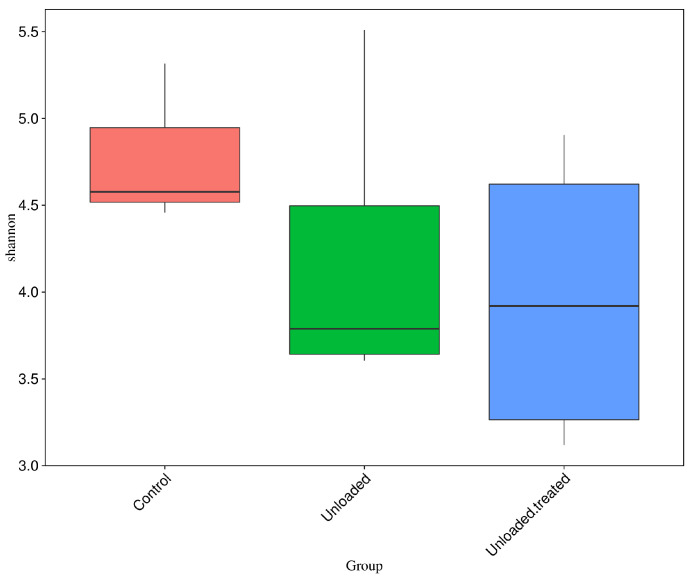
Boxplots for the difference of Shannon indices between control, unloaded, unloaded–treated groups.

Boxplots were formed to analyze the difference in alpha diversity indices between groups. Wilcox and Tukey tests were performed for analysis of the significance of the difference between groups. Boxplots based on the alpha diversity of Shannon indices are shown in Figure 5. The data depict OTU diversity between controls, unloaded mice, and unloaded–treated mice groups.

### 3.8. Community Difference Analysis between Groups: Analysis of Similarity (Anosim)

**Figure 6:**
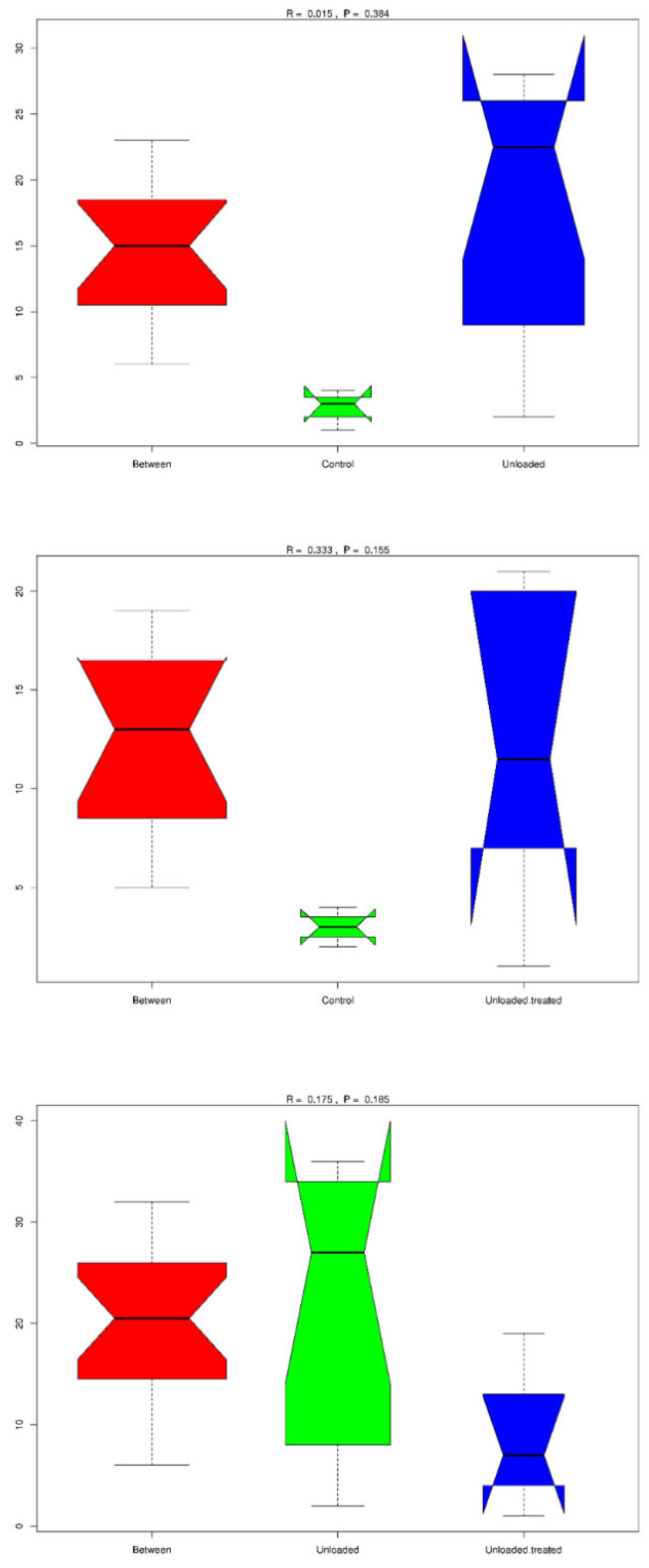
Anosim analysis to evaluate the reasonability of the division of groups. A positive R-value means that inter-group variation is considered significant, while a negative R-value suggests no significant differences. R-value for the control group versus the unloaded group was 0.015; the R value for the control group versus the unloaded-treated group was 0.333; while R-value for the unloaded group versus the unloaded-treated group was 0.175.

Anosim analysis is a nonparametric test to evaluate whether variation among groups is significantly larger than variation within groups, which helps to evaluate the reasonability of the division of groups. According to Anosim results, rank was obtained from the sorted distance between samples. Boxplots based on rank (between the group and within group) are shown in Figure 6. A positive R value means that inter-group variation is considered significant, while a negative R-value suggests that inner-group variation is larger than inter-group variation; therefore, no significant differences were found. R-value for the control group versus the unloaded group was 0.015, R-value for the control group versus the unloaded-treated group was 0.333, while R-value for the unloaded group versus the unloaded-treated group was 0.175.

## 4. Discussion

A plethora of studies is indicative of the profound role of the gut microbiome and its contribution to human health, with dysbiosis of the gut, observed in a variety of metabolic disorders, cancer, and Alzheimer’s disease, to name a few [2,34,35,36]. The gut microbiome performs this role by interacting with the gut lining. The intestinal epithelial cells (IECs) lining the gut ensure the segregation and mediation of intestinal content from systemic circulation, maintain the gut homeostasis [37] and prevent the unwarranted immune responses to gut microbes. The IECs contain highly specialized cell types for these functions, including Paneth cells, goblet cells, enteroendocrine cells, and absorptive epithelial cells [14,38]. Disturbance in the functions of these IECs causes microbial dysbiosis, infiltration, and hyperactivation of immune cells in the lamina propria, contributing to inflammatory bowel disease (IBD). In this regard, we speculated that ER stress and unfolded protein response may contribute to gut dysbiosis, and previous studies have indicated that they play a role in IBD [39,40]. Furthermore, studies have shown that 4-PBA, a potent inhibitor of ER stress, is thought to protect the pancreas, lung, liver, and kidney from injury [41]. Thus, we investigated the therapeutic potential of 4-PBA, an ER stress inhibitor, in the potential restoration of gut dysbiosis using the HU model, a well-recognized ground-based in vivo model of microgravity [12]. The data from our study are indicative that ER stress may be an essential player causing gut dysbiosis that may be transposed by using 4-PBA.

In a healthy adult human, two types of phyla predominate in the gut microbiota are present, namely, Firmicutes (which includes mainly the genera *Enterococcus, Lactobacillus, Clostridium,* and *Faecalibacterium*) and Bacteroidetes (which includes, in particular, the genera *Bacteroides* and *Prevotella*). The other phyla, including Actinobacteria (mainly *Bifidobacterium), Verrucomicrobia, Proteobacteria,* and *Euryarchaeota,* are generally less common than the *Archaea* [42]. As expected, the results from our study revealed that gut dysbiosis was evident after three days using the HU model, as observed in the unloading group [43]. However, treatment with 4-PBA was able to reverse some HU-associated changes in the intestinal microbiome. Our data revealed that the HU model induced changes in the gut microbiome composition of mice. In addition, these perturbed gut bacteria may be associated with changes in gut microbial metabolites, resulting in the disturbance of host metabolite homeostasis causing gut dysbiosis.

Other studies have documented important mucosal microbial changes associated with high-fat food (HFF), such as an increase in Gram-negative bacteria and a corresponding decrease in Gram-positive bacteria. These were associated with phyla-level changes, including a decreased abundance of *Firmicutes* and a relative enrichment in *Bacteroidetes* and *Verrucomicrobia* [44]. Our results revealed that *Bacteroidetes* were the most dominant phyla in the control group of mice, as well as in the unloaded treated with the 4-PBA-treated group of mice. Conversely, *Firmicutes* were the dominant phyla in the unloaded group of mice. Notably, the ratio of *Firmicutes* to *Bacteroidetes* has been investigated previously, and an increase in *Firmicutes* is thought to be concomitant with obesity and other conditions [34,45,46,47]. Our study revealed an increase in *Firmicutes* in the unloaded group as compared to the control group and mice unloaded–treated with 4-PBA (Figure 2), indicating that gut dysbiosis due to an increase in *Firmicutes* to *Bacteroidetes* caused by microgravity may be potentially restored, as observed with the restoration and similar *Firmicutes*-to-*Bacteroidetes* ratio in controls and unloaded–treated mice

Another interesting finding from our study was that the genus *Lachnospiraceae* was higher in relative abundance in unloaded mice in comparison to the unloaded–treated mice and control group. Our data revealed that the relative abundance of this genus was similar in the unloaded–treated and control groups and *Lachnospiraceae* were increased in relative abundance in unloaded mice compared to unloaded–treated mice and controls. Previous work indicates that *Lachnospiraceae* has a controversial role in the human gut [48], influencing health, and some species from this family are increased in abundance during diseases such as metabolic syndrome, obesity, diabetes, liver diseases, and other inflammatory conditions that may be due to *Lachnospiraceae* [49,50].

Likewise, our data revealed that the genus *Akkermansia* was not present in unloaded mice but was observed in controls and unloaded–treated mice. *Akkermansia muciniphila* is considered a beneficial microbe, as well as a promising candidate that has also been suggested as a potential next-generation pre/probiotic [51,52]. Furthermore, *A. muciniphila* is inversely correlated with diabetes, obesity, cardiometabolic diseases, and inflammation [52]. In addition, a variety of pieces of evidence have shown the beneficial impact of this genus in preclinical models [53]. Furthermore, our data revealed that the relative abundance of *Enterorhabdus* was minimal in controls and greatest in relative abundance in unloaded mice. *Enterorhabdus* genus belongs to the Actinobacteria phylum and is thought to be connected with ileocecal mucosal inflammation in mice [54].

Of note, our data showed that microbial diversity was reduced in both 4-PBA-treated and HU mice in comparison to the control group of mice, even though the *Firmicutes*-to-*Bacteroidetes* ratio was restored in the 4-PBA-treated group (Figure 2 and Figure 4). In addition, in the PCoA test, the data indicated that the groups were slightly different in bacterial community structure. Reduced microbial diversity is thought to be associated with gut dysbiosis as well [55], and thus future studies to investigate methods to increase microbial diversity in treated groups should be designed. This may include the addition of pre-/pro- and postbiotic species in the diet of the groups in order to increase microbial diversity, as well as reduce the microbes associated with inflammation, such as *Enterorhabdus*.

Considering the potency of 4-PBA in mitigating ER stress, it appears as an attractive therapeutic candidate to prevent intestinal dysbiosis. To our knowledge, only one study has examined the effects of 4-PBA on intestinal dysbiosis [44]. In this study, mice were fed on HFF exhibited bile acid toxicity and displayed intestinal dysbiosis, which was incompletely reversed with 4-PBA. Several community-level changes in bacterial composition were noted, including an enrichment of Gram-negative bacteria in mice fed on a HFF, and this was prevented by 4-PBA. Moreover, it was observed that a HFF resulted in an increase in bile acid secretion and upregulation of a G-protein-coupled bile acid receptor (TGR5) in Paneth cells. These findings are generally consistent with our study, indicating the therapeutic potential of 4-PBA in preventing intestinal dysbiosis; however, the mechanistic aspects are the subject of future studies and should be explored [44]. ER stress in IECs is caused by two means: (i) IECs are pushed to synthesize copious amounts of proteins, cytokines, and peptides, activating unfolded protein response (UPR). In this outline, cells that are competent enough will survive, and the rest will succumb to stress. (ii) Genetic deficiency of the genes that are involved in UPR, autophagy, secretion, immune response, and inflammation can have various impacts and confer a genetic predisposition to IBD, owing to decreased protein folding capacity and heightened immune response. As discussed earlier, dysfunctional ER stress and UPR is one of the contributing factors in the etiology of IBD [39,40].

Therefore, drugs that target and alleviate ER stress may be a convincing choice to treat IBD or other conditions where there may be gut dysbiosis. Chemical chaperones such as tauroursodeoxycholic acid (TUDCA) and 4-PBA augment protein folding and suppress ER stress [15]. Our results suggest that the reversal of microbial diversity in the HU-treated group might be due to the ER stress inhibitor 4-PBA. We suggest that 4-PBA may eliminate ER stress by targeting receptors on IECs in the hydrophobic regions of the chaperone. This interaction may protect the protein from aggregation, promote the folding of proteins, and reduce ER stress; however, this needs to be ascertained experimentally in future studies. A recent study demonstrated the role of 4-PBA and that ER stress may be involved in the apoptosis of IECs during severe acute pancreatitis [16]. In another recent study, 4-PBA was conjugated with acidic amino acids to yield 4-PBA-glutamic acid (PBA-GA) and 4-PBA-aspartic acid (PBA-AA) conjugates. The study revealed that PBA-GA alleviated damage and inflammation in the colon and substantially reduced the elevated levels of ER stress marker proteins in the inflamed colon in 2,4-dinitrobenzenesulfonic-acid-induced colitis in rats [56].

## 5. Conclusions

To conclude, our data revealed changes in gut microbial diversity in composition in HU mice, indicative of gut dysbiosis. Following treatment with 4-PBA, a potent ER stress inhibitor, the ratio of Firmicutes to Bacteroidetes was restored in unloaded–treated mice, comparable to controls. Furthermore, other beneficial microbes such as *Akkermansia* and *Lactobacillus* were observed in unloaded–treated mice but not in lesser relative abundance in unloaded mice. Our findings suggest that 4-PBA, a potent ER stress inhibitor, may have therapeutic value in treating patients on prolonged bed rest or astronauts during spaceflight.

## Data Availability

Data are available from corresponding author upon reasonable request.

## References

[1] Round J.L., Mazmanian S.K. (2009). The gut microbiota shapes intestinal immune responses during health and disease. Nat. Rev. Immunol..

[2] Goodrich J.K., Waters J.L., Poole A.C., Sutter J.L., Koren O., Blekhman R., Beaumont M., Van Treuren W., Knight R., Bell J.T. (2014). Human genetics shape the gut microbiome. Cell.

[3] Geva-Zatorsky N., Sefik E., Kua L., Pasman L., Tan T.G., Ortiz-Lopez A., Yanortsang T.B., Yang L., Jupp R., Mathis D. (2017). Mining the human gut microbiota for immunomodulatory organisms. Cell.

[4] Magnúsdóttir S., Ravcheev D., de Crécy-Lagard V., Thiele I. (2015). Systematic genome assessment of B-vitamin biosynthesis suggests co-operation among gut microbes. Front. Genet..

[5] Litvak Y., Bäumler A.J. (2019). The founder hypothesis: A basis for microbiota resistance, diversity in taxa carriage, and colonization resistance against pathogens. PLoS Pathog..

[6] Gizard F., Fernandez A., De Vadder F. (2020). Interactions between gut microbiota and skeletal muscle. Nutr. Metab. Insights.

[7] Garrett-Bakelman F.E., Darshi M., Green S.J., Gur R.C., Lin L., Macias B.R., McKenna M.J., Meydan C., Mishra T., Nasrini J. (2019). The NASA Twins Study: A multidimensional analysis of a year-long human spaceflight. Science.

[8] Siddiqui R., Qaisar R., Goswami N., Khan N.A., Elmoselhi A. (2021). Effect of Microgravity Environment on Gut Microbiome and Angiogenesis. Life.

[9] Saei A.A., Barzegari A. (2012). The microbiome: The forgotten organ of the astronaut’s body–probiotics beyond terrestrial limits. Future Microbiol..

[10] Goswami N. (2017). Falls and fall-prevention in older persons: Geriatrics meets spaceflight!. Front. Physiol..

[11] Teodori L., Costa A., Campanella L., Albertini M.C. (2019). Skeletal muscle atrophy in simulated microgravity might be triggered by immune-related microRNAs. Front. Physiol..

[12] Hawliczek A., Brix B., Al Mutawa S., Alsuwaidi H., Du Plessis S., Gao Y., Qaisar R., Siddiqui R., Elmoselhi A.B., Goswami N. (2022). Hind-limb unloading in rodents: Current evidence and perspectives. Acta Astronaut..

[13] Gao Y., Arfat Y., Wang H., Goswami N. (2018). Muscle atrophy induced by mechanical unloading: Mechanisms and potential countermeasures. Front. Physiol..

[14] Ouellette A.J. (2010). Paneth cells and innate mucosal immunity. Curr. Opin. Gastroenterol..

[15] Cao S.S., Zimmermann E.M., Chuang B.M., Song B., Nwokoye A., Wilkinson J.E., Eaton K.A., Kaufman R.J. (2013). The unfolded protein response and chemical chaperones reduce protein misfolding and colitis in mice. Gastroenterology.

[16] You Y.-d., Deng W.-h., Guo W.-y., Zhao L., Mei F.-c., Hong Y.-p., Zhou Y., Yu J., Xu S., Wang W.-x. (2019). 4-Phenylbutyric acid attenuates endoplasmic reticulum stress-mediated intestinal epithelial cell apoptosis in rats with severe acute pancreatitis. Dig. Dis. Sci..

[17] Shkoda A., Ruiz P.A., Daniel H., Kim S.C., Rogler G., Sartor R.B., Haller D. (2007). Interleukin-10 blocked endoplasmic reticulum stress in intestinal epithelial cells: Impact on chronic inflammation. Gastroenterology.

[18] Iannitti T., Palmieri B. (2011). Clinical and experimental applications of sodium phenylbutyrate. Drugs R D.

[19] Zeng M., Sang W., Chen S., Chen R., Zhang H., Xue F., Li Z., Liu Y., Gong Y., Zhang H. (2017). 4-PBA inhibits LPS-induced inflammation through regulating ER stress and autophagy in acute lung injury models. Toxicol. Lett..

[20] Dutta S., Sengupta P. (2016). Men and mice: Relating their ages. Life Sci..

[21] Kovacs G.T., Shadden M. (2017). Analysis of age as a factor in NASA astronaut selection and career landmarks. PLoS ONE.

[22] Baek H., Cho M., Kim S., Hwang H., Song M., Yoo S. (2018). Analysis of length of hospital stay using electronic health records: A statistical and data mining approach. PLoS ONE.

[23] Cao A.-L., Wang L., Chen X., Wang Y.-M., Guo H.-J., Chu S., Liu C., Zhang X.-M., Peng W. (2016). Ursodeoxycholic acid and 4-phenylbutyrate prevent endoplasmic reticulum stress-induced podocyte apoptosis in diabetic nephropathy. Lab. Investig..

[24] Brocca L., Pellegrino M.A., Desaphy J.F., Pierno S., Camerino D.C., Bottinelli R. (2010). Is oxidative stress a cause or consequence of disuse muscle atrophy in mice? A proteomic approach in hindlimb-unloaded mice. Exp. Physiol..

[25] Xia Y., Chen F., Du Y., Liu C., Bu G., Xin Y., Liu B. (2019). A modified SDS-based DNA extraction method from raw soybean. Biosci. Rep..

[26] Youssef N., Sheik C.S., Krumholz L.R., Najar F.Z., Roe B.A., Elshahed M.S. (2009). Comparison of species richness estimates obtained using nearly complete fragments and simulated pyrosequencing-generated fragments in 16S rRNA gene-based environmental surveys. Appl. Environ. Microbiol..

[27] Caporaso J.G., Lauber C.L., Walters W.A., Berg-Lyons D., Lozupone C.A., Turnbaugh P.J., Fierer N., Knight R. (2011). Global patterns of 16S rRNA diversity at a depth of millions of sequences per sample. Proc. Natl. Acad. Sci. USA.

[28] Hess M., Sczyrba A., Egan R., Kim T.-W., Chokhawala H., Schroth G., Luo S., Clark D.S., Chen F., Zhang T. (2011). Metagenomic discovery of biomass-degrading genes and genomes from cow rumen. Science.

[29] Martin M. (2011). Cutadapt removes adapter sequences from high-throughput sequencing reads. EMBnet J..

[30] Quast C., Pruesse E., Yilmaz P., Gerken J., Schweer T., Yarza P., Peplies J., Glöckner F.O. (2012). The SILVA ribosomal RNA gene database project: Improved data processing and web-based tools. Nucleic Acids Res..

[31] Edgar R.C., Haas B.J., Clemente J.C., Quince C., Knight R. (2011). UCHIME improves sensitivity and speed of chimera detection. Bioinformatics.

[32] Haas B., Gevers D., Earl A., Feldgarden M., Ward D., Giannoukos G., Ciulla D., Tabbaa D., Highlander S., Sodergren E. (2011). Chimeric 16S rRNA sequence formation and detection in Sanger and 454-pyrosequenced PCR amplicons. Genome Res..

[33] Edgar R.C. (2004). MUSCLE: Multiple sequence alignment with high accuracy and high throughput. Nucleic Acids Res..

[34] Turnbaugh P.J., Bäckhed F., Fulton L., Gordon J.I. (2008). Diet-induced obesity is linked to marked but reversible alterations in the mouse distal gut microbiome. Cell Host Microbe.

[35] Vogt N.M., Kerby R.L., Dill-McFarland K.A., Harding S.J., Merluzzi A.P., Johnson S.C., Carlsson C.M., Asthana S., Zetterberg H., Blennow K. (2017). Gut microbiome alterations in Alzheimer’s disease. Sci. Rep..

[36] Siddiqui R., Akbar N., Khan N. (2021). Gut microbiome and human health under the space environment. J. Appl. Microbiol..

[37] Okumura R., Takeda K. (2017). Roles of intestinal epithelial cells in the maintenance of gut homeostasis. Exp. Mol. Med..

[38] Peterson L.W., Artis D. (2014). Intestinal epithelial cells: Regulators of Barrier Function and Immune Homeostasis. Nat. Rev. Immunol..

[39] Kaser A., Lee A.-H., Franke A., Glickman J.N., Zeissig S., Tilg H., Nieuwenhuis E.E., Higgins D.E., Schreiber S., Glimcher L.H. (2008). XBP1 links ER stress to intestinal inflammation and confers genetic risk for human inflammatory bowel disease. Cell.

[40] Li A., Song N.-J., Riesenberg B.P., Li Z. (2020). The emerging roles of endoplasmic reticulum stress in balancing immunity and tolerance in health and diseases: Mechanisms and opportunities. Front. Immunol..

[41] Hong Y.-P., Deng W.-H., Guo W.-Y., Shi Q., Zhao L., You Y.-D., Mei F.-C., Zhou Y., Wang C.-Y., Chen C. (2018). Inhibition of endoplasmic reticulum stress by 4-phenylbutyric acid prevents vital organ injury in rat acute pancreatitis. Am. J. Physiol. Gastrointest. Liver Physiol..

[42] Reiss A., Jacobi M., Rusch K., Schwiertz A. (2016). Association of dietary type with fecal microbiota and short chain fatty acids in vegans and omnivores. J. Int. Soc. Microbiota.

[43] Wang Y., Zhao W., Shi J., Wang J., Hao J., Pang X., Huang X., Chen X., Li Y., Jin R. (2019). Intestinal microbiota contributes to altered glucose metabolism in simulated microgravity mouse model. FASEB J..

[44] Zhou H., Zhou S.-Y., Gillilland M., Li J.-Y., Lee A., Gao J., Zhang G., Xu X., Owyang C. (2020). Bile acid toxicity in Paneth cells contributes to gut dysbiosis induced by high-fat feeding. JCI Insight.

[45] Zhang C., Zhang M., Pang X., Zhao Y., Wang L., Zhao L. (2012). Structural resilience of the gut microbiota in adult mice under high-fat dietary perturbations. ISME J..

[46] Guo X., Li J., Tang R., Zhang G., Zeng H., Wood R.J., Liu Z. (2017). High fat diet alters gut microbiota and the expression of paneth cell-antimicrobial peptides preceding changes of circulating inflammatory cytokines. Mediat. Inflamm..

[47] Gomes A.C., Hoffmann C., Mota J.F. (2018). The human gut microbiota: Metabolism and perspective in obesity. Gut Microbes.

[48] Vacca M., Celano G., Calabrese F.M., Portincasa P., Gobbetti M., De Angelis M. (2020). The controversial role of human gut lachnospiraceae. Microorganisms.

[49] Haidar Y.M., Cosman B.C. (2011). Obesity epidemiology. Clin. Colon Rectal Surg..

[50] Festi D., Schiumerini R., Eusebi L.H., Marasco G., Taddia M., Colecchia A. (2014). Gut microbiota and metabolic syndrome. World J. Gastroenterol. WJG.

[51] Voorhies A.A., Mark Ott C., Mehta S., Pierson D.L., Crucian B.E., Feiveson A., Oubre C.M., Torralba M., Moncera K., Zhang Y. (2019). Study of the impact of long-duration space missions at the International Space Station on the astronaut microbiome. Sci. Rep..

[52] Turroni S., Magnani M., Kc P., Lesnik P., Vidal H., Heer M. (2020). Gut microbiome and space travelers’ health: State of the art and possible pro/prebiotic strategies for long-term space missions. Front. Physiol..

[53] Cani P.D., de Vos W.M. (2017). Next-generation beneficial microbes: The case of *Akkermansia muciniphila*. Front. Microbiol..

[54] Yusufu I., Ding K., Smith K., Wankhade U.D., Sahay B., Patterson G.T., Pacholczyk R., Adusumilli S., Hamrick M.W., Hill W.D. (2021). A tryptophan-deficient diet induces gut microbiota dysbiosis and increases systemic inflammation in aged mice. Int. J. Mol. Sci..

[55] Vijay A., Valdes A.M. (2021). Role of the gut microbiome in chronic diseases: A narrative review. Eur. J. Clin. Nutr..

[56] Kim S., Lee S., Lee H., Ju S., Park S., Kwon D., Yoo J.W., Yoon I.S., Min D.S., Jung Y.S. (2020). A colon-targeted prodrug, 4-phenylbutyric acid-glutamic acid conjugate, ameliorates 2, 4-dinitrobenzenesulfonic acid-induced colitis in rats. Pharmaceutics.

